# Optimal Techno-Economic Feasibility of Solar PV Irrigation System Augmented Hydrogen Energy Storage

**DOI:** 10.3390/s26113350

**Published:** 2026-05-25

**Authors:** Mohamed vall O. Mohamed, Turki G. Alghamdi, Farag K. Abo-Elyousr

**Affiliations:** 1 Department of Computer Engineering and Networks, College of Computer and Information Sciences, Jouf University, Sakaka 72388, Saudi Arabia; medvall@ju.edu.sa; 2 Electrical Engineering Department, Faculty of Engineering, Assiut University, Assiut 71516, Egypt; farag@aun.edu.eg

**Keywords:** energy system management, water pumping PV, irrigation, water demand, techno-economic

## Abstract

To deliver freshwater for drip irrigation, our study presents an optimal techno-economic based on a Water Pumping Photovoltaic System (WPPVS) that integrates a Hydrogen Energy Storage System (HySS) to ensure reliable freshwater for agricultural irrigation in remote arid regions. A critical operational challenge in WPPVS is mechanical vibration at low flow rates, which degrades the pump efficiency and lifespan. Our methodology directly addresses this issue by incorporating a vibration-avoidance strategy that ensures that the pump operates only within its stable and, efficient range. To reduce the loss of water supply probability and overall annual costs of the drip irrigation system, a multi-objective optimization framework using Multi-Objective Particle Swarm Optimization (MOPSO) and Gaussian Mixture Model (GMM) clustering to simultaneously minimize the Loss of Water Supply Probability (LWSP), and the system’s total life-cycle cost. The model’s practical applicability is demonstrated through a detailed techno-economic feasibility analysis for a tomato crop drip irrigation project in Sakaka, Saudi Arabia. Sensitivity analysis is performed on dynamic head, crop prices, and interest and inflation rates, confirming the robustness of the system against variable economic indicators. In comparison to 1071 h without HySS, the results revealed that the seasonal irradiation harvest hours are 1863, which represents 21% of the seasonal hours employing the developed hybrid energy storage coordination. This integrated approach provides a holistic and economically viable solution for designing reliable solar irrigation systems with long-term mechanical integrity.

## 1. Introduction

### 1.1. Motivation

Cost-effective, sustainable, and reliable energy—particularly for agricultural irrigation—remains unavailable in many isolated and developing regions [1]. RES are considered a viable solution due to their technological maturity, economic feasibility and environmental benefits, as well as their widespread availability [2]. In this context, WPPVS facilitate the integration of solar energy into irrigation applications [3], supporting the development of modern and decentralized energy systems. In such systems, energy is generated close to the point of use through PV technologies with varying capacities and configurations. This reduces infrastructure requirements and minimizes transmission and distribution losses. In addition, the incorporation of energy storage systems improves the resilience of WPPVS by allowing improved adaptability to dynamic operating conditions and environmental variability. These features are essential to ensure long-term performance under impacts of climate change. However, the increasing flexibility and scalability of modern power distribution networks introduce additional challenges in microgrid design. Furthermore, WPPVS are susceptible to mechanical vibrations at low flow rates, which may adversely affect system efficiency and operational lifetime.

Several factors complicate the optimal design and operation of WPPVS, particularly in the sizing of PV systems and associated storage components. These aspects include: (i) The availability of renewable energy is strongly influenced by economic conditions, weather variability, and the complementary characteristics of different RES. (ii) Although PV systems can generate substantial energy [4], their intermittent nature necessitates appropriate storage solutions, such as reservoirs, to maintain system continuity during periods without solar irradiation [5]. (iii) Irrigation requirements highlight the need for effective vibration mitigation strategies to ensure safe pump operation under low dynamic head conditions [3]. (iv) The integration of advanced storage technologies, including batteries, pumped hydro-storage, and Hydrogen Energy Storage System HySS, introduces challenges related to operational and lifecycle costs. This requires the application of multi-objective optimization techniques to achieve balanced system performance. (v) There is a need for forward-looking design and validation approaches for water irrigation and WPPVS applications. (vi) Energy consumption has become complex. Modern irrigation systems used for various crops complicate planning and operations due to fluctuating energy demand and generation.

Motivated by these challenges, this study proposes a WPPVS integrated with a hybrid storage system comprising a water storage tank and a HySS to ensure sustainable long-term operation. The proposed configuration aims to reduce total system cost, enhance energy management, and decrease the loss of water supply probability, while also mitigating pump vibrations. To address these objectives, a multi-objective optimization framework is developed using Multi-objective Particle Swarm Optimization (MOPSO) combined with Gaussian Mixture Model (GMM) clustering. The proposed approach simultaneously minimizes total cost and loss of water supply probability while incorporating a vibration avoidance strategy. The effectiveness of the framework is demonstrated through a case study of a drip irrigation project in Sakaka, Al Jouf Region, Saudi Arabia.

### 1.2. Related Works

The rapid development of hybrid renewable energy systems (HySS) and renewable-energy-driven technologies has been widely reported as a sustainable solution to environmental and energy challenges. Studies such as [6] highlight the integration of photovoltaic (PV), wind, and biomass-based systems in electrochemical and water-treatment applications, particularly for remote and off-grid areas. Despite their potential, challenges related to intermittency, scalability, and economic feasibility remain significant [7,8]. In parallel, photovoltaic water pumping systems (WPPVS) have been demonstrated as technically and economically viable solutions for irrigation and rural water supply, encouraging the use of intelligent and metaheuristic-based optimization methods for system sizing and performance improvement [9,10,11].

Recent research has increasingly focused on multi-objective optimization and intelligent energy management in hybrid renewable systems. For example, the framework proposed in [12] combines Taguchi design, moth-flame optimization, and fuzzy decision-making to reduce energy costs and improve reliability, outperforming conventional techniques such as NSGA-II and MOPSO. Similarly, hybrid PV–wind–diesel systems optimized using evolutionary algorithms have shown improvements in lifecycle cost, emissions reduction, and system reliability [13,14]. Advanced control strategies, including fuzzy logic and hybrid maximum power point tracking (MPPT), further enhance system robustness and reduce battery degradation under variable environmental conditions [15]. However, most of these studies focus on general hybrid microgrids, while applications of HySS specifically tailored for irrigation systems remain limited.

A substantial body of work has addressed PV-based WPPVS for irrigation. Socio-economic and experimental analyses confirm their feasibility and effectiveness in reducing diesel consumption and operational costs [16,17]. Advanced techniques such as hydraulic power point tracking and vibration-aware operation have been shown to significantly improve water delivery and system efficiency under varying irradiation conditions [3,18]. Techno-economic studies across different regions further demonstrate that solar irrigation systems achieve competitive levelized costs and short payback periods compared to conventional systems [19,20,21]. Nevertheless, most existing studies treat electrical and hydraulic subsystems independently, limiting accurate modeling of their coupled dynamic behavior.

Hybrid configurations have been explored to enhance system reliability and mitigate renewable energy intermittency. PV–wind hybrid pumping systems have demonstrated improved water output compared to standalone systems [22], while artificial intelligence-based energy management approaches improve forecasting accuracy and operational efficiency [23]. Additional configurations incorporating storage or hybrid energy management strategies further enhance the stability and resilience of the system [24,25]. However, these approaches are largely energy-centric and rarely account for hydraulic constraints such as Best Efficiency Point (BEP) deviation, cavitation, and vibration, which are critical for irrigation system performance.

Pump operation outside the BEP remains a major technical challenge in WPPVS, leading to instability, cavitation, and reduced system lifespan [26,27]. Although mitigation strategies such as vibration avoidance and data-driven diagnostics have been proposed [28,29], they are typically not integrated into renewable energy optimization frameworks. This highlights a disconnect between hydraulic reliability analysis and energy system design in existing studies.

HySS have recently gained attention for rural electrification and resilient microgrid applications, with promising system readiness levels and improved economic performance through advanced control strategies such as model predictive control [30,31]. However, challenges such as battery degradation, hydrogen safety risks, and system complexity persist [32]. More importantly, the application of HySS to irrigation systems remains scarce, particularly in frameworks that integrate multi-objective optimization with detailed hydraulic modeling and operational constraints.

Finally, it can be observed that the existing literature reveals a clear gap between advancements in hybrid renewable energy systems and their application to irrigation. While significant progress has been made in energy optimization and PV-based pumping technologies, few studies address the combined challenges of HySS integration, energy–hydraulic coupling, and operational reliability in irrigation systems. This gap motivates the present work, which employs multi-objective particle swarm optimization (MOPSO) to develop an integrated and optimized WPPVS framework for irrigation applications.

### 1.3. Research Gaps and Contributions of the Paper

#### 1.3.1. Research Gap

The evaluation of the literature as shown in Table 1 highlights certain research gaps that need to be tackled: (i) Despite the several examples of WPPVS paired with PV, further research is necessary to resolve vibration difficulties. (ii) The weather conditions, including temperature, wind speed, pressure, humidity, rainfall and hourly solar radiation, are thoroughly considered when calculating the LWSP along with minimizing overall costs. Therefore, obtaining the best or optimal solution through conventional optimization techniques becomes difficult. (iii) Thorough consideration of restrictions on the multi-objective optimization technique, such as rainfall, water level in the well, water requirements, and maximum pumped flow rate.

This study aims to develop a reliable EMS optimization technique that considers each of the considerations mentioned above. The developed WPPVS seeks to satisfy cost considerations while adhering to all LWSP-related restrictions. Therefore, the major contributions of the current study are:

- A WPPVS integrated with vibration avoidance technique is developed for drip irrigation systems in the vast desert regions of Saudi Arabia, particularly the Sakaka area in the northern part of the country, due to the scarcity of surface water.
- Developing optimization techniques based on MOPSO in order to uncover the nonlinear aspects of the WPPVS integrated drip irrigation system’s global optimum. This can be especially useful when addressing an issue that may have several local solutions that can be implemented. GMM, therefore clustering is used to determine the near-optimal sizes for the storage tank, PV generator, and HySS.
- As a part of the drip irrigation system, developing a hybrid ESS that combines a water tank with a HySS, making the installation of solar PV more affordable. Therefore, whether this energy can be used to store hydrogen could have a positive impact on the energy market.

#### 1.3.2. Contributions of the Paper

A variety of technical information and approaches have been used to address WPPVS in various microgrids are provided in Table 1. A review of the references listed in Table 1 demonstrates that recent studies have mainly focused on improving solar energy harvesting PV through ready-made software or analytical methodologies. The former provides a black box data entry, whereas the latter emphasizes a single objective, requiring additional work for multi-objective functions. Furthermore, research into the role of storage systems such as HySS is rare in the literature, particularly when tackling fundamental but unusual concerns such as the threat of a water supply outage in determining the optimal decision variables. To the best of authors’ knowledge, avoiding vibrations in WPPVS irrigation systems that integrate a storage tank and HySS is infrequently described in the literature. The main goals of the current study are stated below to provide a feasible and economic storage and generation of a WPPVS:

- To develop a platform model that reduces water supply failures and costs while preventing the pump vibrations and the energy waste during operation.
- To assess the WPPVS’s long-term performance using a hybrid energy storage system comprising a tank and a HySS.

### 1.4. Paper Organization

The remainder of the structure of this paper is organized as follows. Section 2 discusses the problem description and modeling of the system and provides a comprehensive mathematical framework. The Simulation results are presented in Section 3. Finally, the Conclusions and Perspectives are provided in Section 4.

## 2. Problem Description and System Modeling

**Figure 1:**
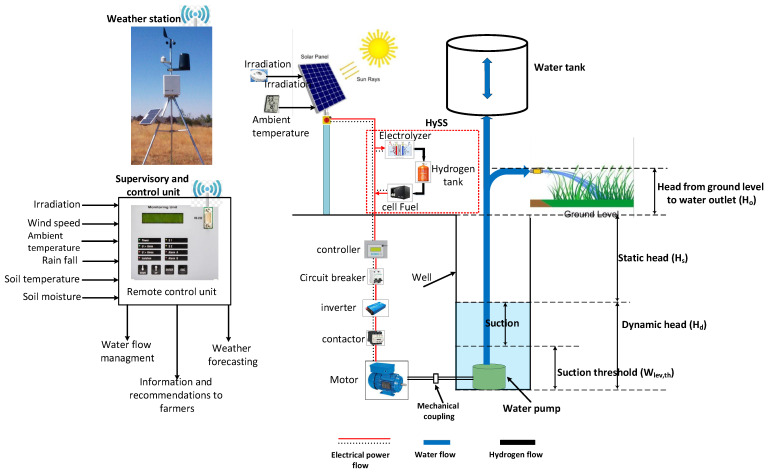
Architecture of the irrigating WPPVS with HySS sizing.

Solar PV module is used in the proposed WPPVS to irrigate fields using solar energy. As shown in Figure 1, an inverter is used to extract electrical power to pump underground water through the motor and mechanical coupling. The output voltage of solar PV module is controlled by a PV controller, contactor, and circuit breaker. This WPPVS is used to irrigate two hectares of tomato crops in Sakaka, Aljouf, which is located in the northern part of Saudi Arabia at latitude 29.9° N and longitude 40.2° E [3]. The tomato season begins in this area at the beginning of September and culminates in the first week of February, lasting 153 days of growth. The inverter’s output voltage drives a motor. The pump serves to supply the tomato field according to the EMS, which is mainly dependent on solar irradiation. The injected water is used to irrigate the crop and refill the tanks. However, due to the vibration avoidance strategy, HySS is employed to store energy when the pump is not in operation. Implementing a monitoring and controlling module for the irrigation process via IoT techniques is necessary for minimizing water usage when using modern irrigation techniques like drip or sprinkler instead of traditional surface irrigation. Based on meteorological factors (temperature, solar radiation, humidity, etc.), it aids in estimating the crops’ water requirements. By utilizing information and communication technologies to incorporate climatic data into agricultural decision-making processes, this sensor-based system offers an information platform that helps farmers make the best use of water and increase crop output. As a result, farmers are better able to adjust to new agricultural technologies.

### 2.1. System Modeling

#### 2.1.1. PV Modeling

The solar PV output power in kW is given as (1) in terms of solar irradiation $G_{n}$ and ambient temperature $T_{air}$.

(1) $$\begin{array}{c} \begin{array}{c} P_{pv}\left(t\right)=f_{PV}\;P_{PV\_rat}[1+\beta_{r}\left(T_{c}\left(t\right)-T_{r}\right)]\frac{G_{n}\left(t\right)}{G_{r}} \end{array} \end{array}$$

(2) $$\begin{array}{c} \begin{array}{c} T_{c}\left(t\right)=T_{a}\left(t\right)+\frac{NOCT-20}{0.8}G_{n}\left(t\right) \end{array} \end{array}$$

where, $P_{PV\_rat}$ is the rating of the PV module at $G_{r}$ is 1kWh/m^2^ and $T_{r}$ is 25°, respectively. $f_{PV}$ is the derating factor (0.98), which considers several losses such as aging, wiring, impact of soiling on modules, snow cover, and shading. $\beta_{r}$ (−3.7 × 10^3^ /°C) is the thermal coefficient of solar cells. $G_{n}$ is Solar irradiation in (kWh/m^2^). NOCT is the “nominal cell operating temperature” at $G_{n}$ of 1 kWh/m^2^ and $T_{r}$ of 25 °C. The parameter maintains a nominal value of approximately 44 ± 2 °C. All system parameters, nomenclature, and symbols are defined in the Supplementary Materials. Herein, the key finding is to optimally find $P_{PV\_rat}$ to meet the pareto front of multi-objective functions.

#### 2.1.2. Hydraulic Pump Modeling

The water flow rate $Q_{d}$ and the total dynamic head $H_{t}$ are used to compute the pump output power $P_{hdro}$ as:

(3) $$\begin{array}{c} \begin{array}{c} P_{hdro}=\rho \;g\;Q_{d}\;H_{t} \end{array} \end{array}$$

(4) $$\begin{array}{c} \begin{array}{c} H_{t}=H_{d}+H_{s}+H_{o} \end{array} \end{array}$$

where, ρ is the density of water (kg/m^3^) and *g* is the gravitational field strength (N/kg). $H_{d}$ is the dynamic head as in Figure 1, $H_{s}$ is the static head, and $H_{o}$ is the head at the ground level relative to the outlet of the water above the ground, all given in meters, respectively. The required PV power is given in terms of the pump and inverter efficiencies as:

(5) $$P_{pv}=\frac{P_{hdro}}{\eta_{p}\;\eta_{inv}}$$

where, $\eta_{p}$ is the pump efficiency, which includes hydraulic, mechanical losses, and the coupled motor efficiency in the pumping mode, $\eta_{inv}$ is the inverter efficiency (DC–AC conversion efficiency). Combining (1), (3) and (5), the water flow rate (m^3^/h) is estimated as follows:

(6) $$Q_{d}\left(t\right)=\frac{P_{pv}\left(t\right)\;\eta_{p}\;\eta_{inv}}{\rho \;g\;H_{t}}$$

#### 2.1.3. HySS Modeling

In the current WPPVS, energy storage is accomplished by the use of the HySS in times of excess energy. This energy is converted into hydrogen by the electrolyzer and then stored in the hydrogen tanks. When necessary, the fuel cell converts this hydrogen into electrical power. In turn, the energy output of the fuel cell ($E_{fc}$) at time *t* is [30]:

(7) $$E_{fc}\left(t\right)=37.8\;H_{2u}\;\eta_{FC}$$

where, $H_{2u}$ is the hydrogen used in kg, $\eta_{FC}$ is the efficiency of the fuel cell ($\eta_{FC}$ = 0.5). In (8), where $\eta_{elec}$ is the efficiency of the electrolyzer ($\eta_{elec}$ = 0.7) and $P_{r}$ is the PV power portion used to produce hydrogen, the hydrogen produced by the electrolyzer $H_{2p}$ is:

(8) $$H_{2p}\left(t\right)=\frac{\eta_{elec}\;P_{r}\left(t\right)}{37.8}$$

As demonstrated in (9), the hydrogen in the tank accumulates when the PV energy exceeds the load needs.

(9) $$H_{2t}\left(t\right)=H_{2t}\left(t-1\right)+H_{2p}\left(t\right)$$

where, $H_{2t}$ is the hydrogen inside the tank. Yet, if the PV energy output is insufficient to meet the load demands, the hydrogen level of the tank is modified using (9).

(10) $$H_{2t}\left(t\right)=H_{2t}\left(t-1\right)-H_{2u}\left(t\right)$$

The following limitations regulate the amount of hydrogen in the tanks.

(11) $$\begin{array}{c} \begin{array}{c} H_{2tmin}\leq H_{2t}\left(t\right)\leq H_{2tmax} \end{array} \end{array}$$

(12) $$\begin{array}{c} \begin{array}{c} H_{2tmin}=0.2\;H_{2tmax} \end{array} \end{array}$$

(13) $$\begin{array}{c} \begin{array}{c} H_{2tmax}=N_{t}\;H_{2nom} \end{array} \end{array}$$

where, $H_{2tmin}$ and $H_{2tmax}$ are the maximum and minimum hydrogen levels of the tank, respectively, $H_{2nom}$ are the nominal storage capacity of the tank in kWh and $N_{t}$ is the number of tanks. Herein, the key findings are to size up the optimal number of hydrogen tanks, fuel cell output power, and number of electrolyzers to meet a multi-objective function.

### 2.2. Inverter Modeling

The inverter is designed to handle the maximum expected power ($P_{max}$) from the load demands. With $\eta_{inv}$ representing the efficiency of the inverter, the inverter power rating ($P_{inv}$) is:

(14) $$P_{inv}=\frac{P_{max}}{\eta_{inv}}$$

### 2.3. Water Necessary for Irrigation

According to [3], the hourly reference evapotranspiration ($E_{ref}$) in mm/h that is taken from FAO can be written as:

(15) $$E_{ref}\left(t\right)=\frac{0.113\;\Delta \;\left(G_{n}\left(t\right)-G\left(t\right)\right)+\gamma \left(900/T_{a}\left(t\right)+273\right))\;v_{w}\left(t\right)\;\left(e_{s}\left(t\right)-e_{a}\left(t\right)\right)}{\Delta +\gamma (1+0.34v_{w}\left(t\right))}$$

where, *G* is the soil ’heat flux density’ in kWh/m^2^ on an hourly basis and $G_{n}$ is the solar irradiation as defined in Section 2.1.1. $v_{w}$ is the wind speed at 2m height from the ground in m/s. During the day, *G* equals 0.1 $G_{n}$, whereas at night, it equals 0.5 $G_{n}$. The parameter Δ in kPa/°C is given in terms of air temperature $T_{a}$ as:

(16) $$\Delta =\frac{4098\;e_{s}}{{\left(T_{a}\left(t\right)+273.3\right)}^{2}}$$

γ in kPa/°C is expressed as (17), in which $P_{a}$ is the atmospheric pressure in kPa/°C. In addition, $e_{s}$, which takes into account the saturation vapor pressure, is estimated as in (18).

(17) $$\gamma =0.665\times 10^{-3}P_{a}$$

(18) $$e_{s}=0.6108\;e^{\frac{17.27\;T_{a}\left(t\right)}{T_{a}\left(t\right)+273.3}}$$

$e_{a}$, which considers the actual hourly vapor pressure, is expressed in (19).

(19) $$e_{a}=0.01\;e_{S}\;H_{r}$$

$H_{r}$ is the relative humidity. $E_{c}$, which is the actual evapotranspiration per hour in m^3^/h is determined by multiplying the value of $E_{ref}$ by the crop coefficient $K_{c}$ to consider the features of the crop as follows. In tomato crops, $K_{c}$ values are of 0.6, 1.52, and 0.7 at the starting, middle, and last stages of the season, respectively [46].

(20) $$E_{c}\left(t\right)=20\;K_{c}\;E_{ref}\left(t\right)$$

$E_{c}$ helps maintain the equilibrium of soil moisture ($M_{s}$). The soil moisture in m^3^/h is given in (21), in which $Q_{d}$ is the amount of water that is delivered to the planted region and $P_{pre}$ is the precipitation in m^3^/h. $P_{pre}$ has values of 8 × 10^−3^, 75 × 10^−3^, and 116 × 10^−3^ at the starting, middle, and final stages of the season, respectively. $P_{rain}$ is related to rain fall $P_{rnfall}$ in m^3^/h at an hour *t* as in (22) [3].

(21) $$M_{s}\left(t\right)=M_{s}\left(t-1\right)+Q_{d}\left(t\right)+P_{pre}\left(t\right)+P_{rain}\left(t\right)-E_{c}\left(t\right)$$

(22) $$P_{rain}\left(t\right)=\left\{\begin{array}{ccc} 16\;P_{rnfall}\left(t\right)-0.7; & \;: & P_{rnfall}\left(t\right)\;>2.08\\ 12\;P_{rnfall}\left(t\right)-0.28 & \;: & P_{rnfall}\;\leq 2.08 \end{array}\right.$$

In addition, both $E_{c}$ and $M_{s}$ define the crop yield in (ton/ha). Equation (23) is used to determine the real yield ($Y_{a}$) from the injected water.

(23) $$Y_{a}\left(t\right)=Y_{m}\left(1-K_{y}\frac{M_{s}\left(t\right)}{E_{c}\left(t\right)}\right)$$

where, $Y_{m}$ is the maximum crop production of value 36.6 ton/ha and $K_{y}$ is the “the yield response factor” of value 1.05 [47].

### 2.4. Economic Analysis

In WPPVS, the seasonal pay-off (profit) is the subtraction of the entire cost of the WPPVS and the seasonal expenses of the agricultural process from the seasonal revenue from tomato production as (24), in which every component is given in $/kWh.

(24) $$C_{payof}\left(t\right)=C_{in}\left(t\right)-C_{sys}\left(t\right)-C_{agr}\left(t\right)$$

where, $C_{payof}$ is the seasonal profit, $C_{in}$ is the seasonal revenue or income, $C_{sys}$ is the WPPVS component costs per season, and $C_{agr}$ is the seasonal agricultural costs. According to estimate, workers, machinery, and production necessities (such as the cost of seeds, fertilizer, and pesticides), $C_{agr}$ accounted for 49% and 21% of the tomato crop’s overall expenditures, respectively.

The actual crop yield in (23) for an irrigation area (*A*) determines the seasonal revenue $C_{in}$ from the tomato sale as:

(25) $$C_{in}\left(t\right)=P_{t}\;A\;Y_{a}$$

where, $P_{t}$ is the tomato market price ($/ton) and *A* is the irrigated area in ha. The $C_{sys}$ is related to the Net Present Costs of each component ($NPC_{c}$) as:

(26) $$NPC_{c}=C_{ic}+C_{repc}+C_{omc}-C_{salvc}-C_{fc}$$

where, $C_{ic}$ is the initial cost, $C_{repc}$ is the replacement cost, $C_{omc}$ is the operation and maintenance costs, and $C_{salvc}$ is the salvage cost, and $C_{fc}$ is the fuel cost, respectively. The $C_{ic}$ is given as in (27), in which $N_{c}$ is the number of components, $P_{nc}$ is the nominal rating of each component, and $C_{com}$ is the initial capital cost of each component. All costs are in $/kW.

(27) $$C_{ic}=N_{c}\;P_{nc}\;C_{com}$$

The replacement costs are given as:

(28) $$C_{repc}=C_{ic}\sum_{k=1}^{K_{rep}}\frac{1}{{\left(1+i\right)}^{LF_{c}\times k}}$$

where, $LF_{c}$ is the lifetime of each component and *i* is the interest rate, which is estimated based on the inflation rate IF as,

(29) $$i=i_{n}-IF$$

The number of replacements $K_{rep}$ is determined in terms of the project lifetime (LF) as.

(30) $$K_{rep}=\left\{\begin{array}{ccc} \frac{LF}{LF_{c}}-1; & \;: & \frac{LF}{LF_{c}}\;=0.0\\ floor(\frac{LF}{LF_{c}}) & \;: & \frac{LF}{LF_{c}}\;\neq 0.0 \end{array}\right.$$

where, the floor function refers to the lower limit of the resulting figure. Based on the notable shift in interest rates and the corresponding increase in worker wages in emerging countries, the literature estimated that operating and maintenance costs would range from 5% to 10% [3,30]. The value of each salvaged component at the end of the project is based on the number of years it will remain functional [3]. Thus, it is a fraction of the original cost of the component as:

(31) $$C_{salvc}=\left\{\begin{array}{ccc} 0.1\times C_{ic}\;\frac{1}{{\left(1+i\right)}^{LF}}; & \;: & \frac{LF}{LF_{c}}\;=0.0\\ C_{ic}\frac{Rem_{yc}}{LF_{c}}\;\frac{1}{{\left(1+i\right)}^{LF}} & \;: & \frac{LF}{LF_{c}}\;\neq 0.0 \end{array}\right.$$

where, $Rem_{yc}$ is the remaining lifetime of a component and $LF/LF_{c}$ determines the number of replacements for a component. $LF/LF_{c}$ influences the remaining lifetime of the as:

(32) $$Rem_{yc}=LF_{c}+\left(LF-floor\left(\frac{LF}{LF_{c}}\right)\right)$$

Thus, considering the whole number of components $N_{c}$, the WPPVS system component costs per season are impacted by the NPC in the following ways:

(33) $$C_{sys}\left(t\right)=\sum_{k=1}^{N_{c}}NPC_{k}\times CRF$$

where,

(34) $$CRF=\frac{i{\left(i+1\right)}^{LF}}{{\left(1+i\right)}^{LF}-1}$$

### 2.5. Avoidance of Pump Vibrations Due to Changes in Flow Rate

Due to their extreme sensitivity to changes in solar radiation, WPPVS experience variations in input power and, as a result, pump flow rate. Increased vibrations from these flow rate variations can lead to unstable pump operation, which lowers efficiency, jeopardizes reliability, and shortens pump service life. Preventing the pump with the help of ESS from running in areas of low flow and low efficiency, where mechanical vibrations and energy losses are the most severe, is one of the main goals of this research [3].

**Figure 2:**
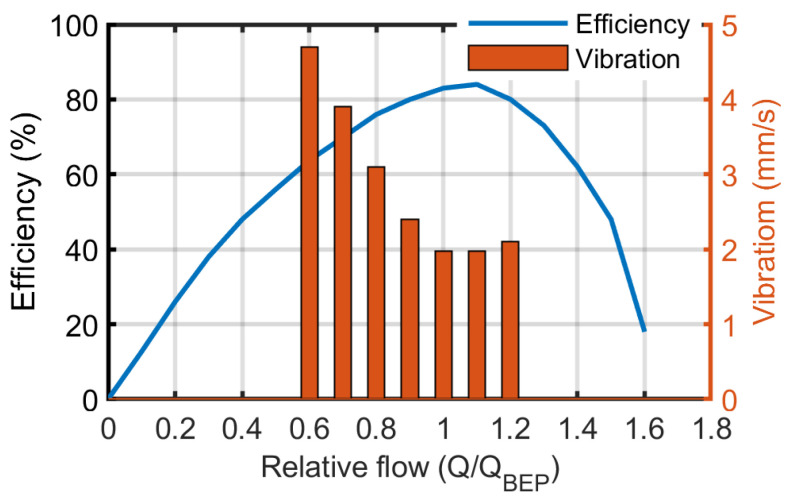
Vibration and efficiency in relation to relative water flow [26].

Figure 2 illustrates the relationship between pump vibration in (mm/s), hydraulic efficiency, and the relative flow rate (*Q*/$Q_{BEP}$), where $Q_{BEP}$ represents the water flow at the BEP. At relative flow values below 0.6, the pump experiences high vibration and low efficiency, making this range inappropriate for operation. For relative flow rates ranging from 0.6 to 1, both vibration and efficiency are initially moderate and progressively improve. The optimal operating condition occurs at the BEP (relative flow = 1), where vibration is minimized at 1.9 mm/s and efficiency is maximized at 83.3%.

The vibration threshold (relative flow < 0.6) is not merely a theoretical constant [3]; it represents a critical signal-to-noise transition point captured by the accelerometer. The raw vibration data is processed using the Fourier transform of the time series data [26] to identify peak frequencies. In this study, because the relative flow rate is straightforward to estimate, it is utilized to avoid the pump vibration instead of analyzing the data via Fourier transform. When the relative flow drops below 0.6, the sensor detected a significant increase in mechanical resonance, which serves as a trigger for system recalibration. This approach ensures that the model responds to real physical disturbances rather than ideal inputs.

### 2.6. Multi-Objective PSO

Particle Swarm Optimization (PSO) simulates the social behavior of bird flocks or fish schools to explore complex search spaces. In PSO, a swarm of particles (candidate solutions) moves through the solution space by adjusting their velocities and positions based on both their individual best-known positions and the global best-known position found by the swarm. The speed of the swarm (*v*) changes the position (*X*) of a particle (*i*) at iteration *t*, similar to (35) and (36), where (*GB*) denotes the global best and (*PB*) represents a leader best. Random assumption is used for additional elements. Ultimately, the optimal choice is recorded at each iteration.

(35) $$\begin{array}{c} \begin{array}{c} v_{t}^{i}=\omega_{0}v_{t}^{i-1}+r_{1}c_{1}\left(GB^{i}-X_{t}^{i}\right)+r_{2}c_{2}\left(PB-X_{t}^{i}\right) \end{array} \end{array}$$

(36) $$\begin{array}{c} \begin{array}{c} X_{t}^{i+1}=X_{t}^{i}+v_{t}^{i} \end{array} \end{array}$$

**Figure 3:**
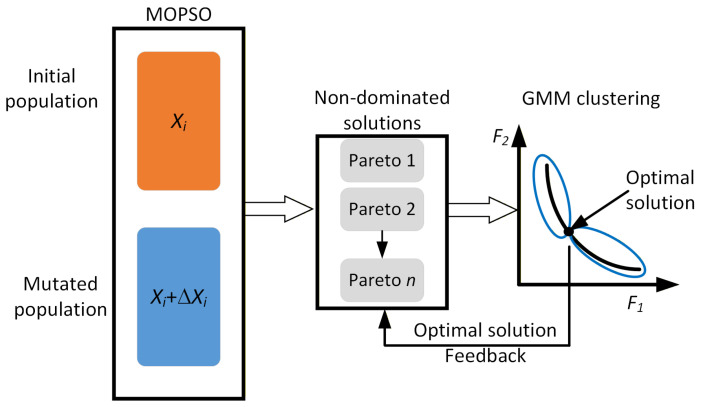
Gaussian mixture model clustering-based MOPSO.

This process allows the algorithm to balance exploration and exploitation, making it suitable for solving nonlinear and multimodal optimization problems. By keeping an external archive of non-dominated solutions that approximate the Pareto front, as shown in Figure 3, MOPSO expands PSO to handle optimization problems with two or more competing objectives. MOPSO includes mechanisms for preserving the diversity between solutions, such as crowding distance or clustering in the objective space, to prevent premature convergence and ensure a wide spread of trade-offs. The following procedures are used to explain MOPSO [48]:

1. Randomly, set the population, $X_{i}$, to its initial value for *i* = 1, 2,…, n, where n is the number of the population.
2. Make the initial positions $X^{i}$ the global best locations found so far.
3. For every particle, set the initial velocity, $v_{j}$, to zero. Subsequently, each particle is assessed as in (36).
4. Such a multiobjective function yields non-dominated solutions. The non-dominated solutions and their corresponding objectives are then archived in an array.
5. Estimate the speed of each particle as in (35), from which the positions of each particle are updated as in (36).
6. Every particle, in this step, undergoes mutation, which is a random perturbation added to the particle’s position. In addition, the particles are kept within the search space.
7. Adjust the position of each particle by replacing its current best position with the previous best position.
8. Demonstrate the resulting Pareto front whenever the maximum iteration has been completed.

#### 2.6.1. Gaussian Mixture Model Clustering Based on Multi-Objective Particle Swarm Optimization

The GMM are widely used probabilistic models in unsupervised learning that assume data points are generated from a mixture of multiple Gaussian distributions, each representing a different cluster [49]. Figure 3 shows the process diagram for the GMM based MOPSO. After being extracted from MOPSO, the Pareto front is placed in GMM, where it is clustered.

Each Gaussian component is defined by a mean vector and a covariance matrix, and the mixture is governed by a set of weights that represent the prior probability of each component. The key strength of GMM lies in their flexibility to model complex data distributions, including elliptical and overlapping clusters. GMM use a soft probabilistic approach to group data points into clusters, giving each point a membership probability for every cluster. This allows for more nuanced modeling, especially in situations where cluster boundaries are not clearly separable. Parameter estimation in GMMs is typically performed using the Expectation-Maximization algorithm, which iteratively updates the parameters to maximize the likelihood function.

#### 2.6.2. Knee-Point Selection Strategy for Pareto Optimal Decision Making

In this work, the input to the GMM consists of the Pareto-optimal solution set generated by the MOPSO, defined as $X={\left\{\left(COE_{i},LWSP_{i}\right)\right\}}_{i=1}^{N}$, where each solution is represented in a two-dimensional objective space comprising the seasonal costs of energy and the LWSP. This formulation allows the clustering process to directly capture the trade-off relationship between economic and performance objectives. The number of clusters is set to K=2 based on the observation of two dominant trade-off regions in the Pareto front (low-cost and high-performance solutions), and is further supported by silhouette score evaluation.

In multi-objective optimization problems, the Pareto front typically consists of a set of non-dominated solutions representing different trade-offs between conflicting objectives [50]. However, for practical engineering implementation, a single compromise solution is often required. In this study, a knee-point based selection strategy is adopted to identify the most balanced operating point of the WPPVS from the obtained Pareto front [51]. The knee point is defined as the solution where a marginal improvement in one objective results in a disproportionately large degradation in at least one other objective [52]. Mathematically, this corresponds to the region of maximum curvature along the Pareto front in the normalized objective space. Let the normalized objective vector be defined as:

(37) $$F\left(x\right)=\left[f_{1}\left(x\right),f_{2}\left(x\right)\right],$$

where $f_{1}\left(x\right)$ represents the total seasonal cost and $f_{2}\left(x\right)$ represents the LWSP, both normalized to the range [0,1] to ensure scale independence.

To identify the knee point, the curvature κ of the Pareto front is evaluated using consecutive solution points. For three successive points $F_{i-1},F_{i},F_{i+1}$, the curvature is approximated as:

(38) $$\kappa_{i}=\frac{\left|\left(F_{i}-F_{i-1}\right)\times \left(F_{i+1}-F_{i}\right)\right|}{∥F_{i}-F_{i-1}∥∥F_{i+1}-F_{i}∥∥F_{i+1}-F_{i-1}∥},$$

where × denotes the scalar cross product in the two-dimensional objective space. The knee point is then selected as:

(39) $$x^{*}=\arg\max_{i}\;\kappa_{i}.$$

which is consistent with curvature-based knee detection approaches in multi-objective optimization [51,53].

This formulation ensures that the selected solution lies at the region of maximum trade-off intensity, where any further improvement in one objective would lead to a significant deterioration in the other. To enhance robustness and avoid sensitivity to local fluctuations in the Pareto front, a GMM is further integrated into the selection process. Let the Pareto solutions be modeled as a mixture distribution:

(40) $$p\left(F\right)=\sum_{k=1}^{K}\pi_{k}\;N\left(F|\mu_{k},\Sigma_{k}\right),$$

where $\pi_{k}$ are the mixture weights, and N(·) represents the Gaussian distribution with mean $\mu_{k}$ and covariance $\Sigma_{k}$. The clustering process partitions the Pareto front into distinct operating regimes.

In this context, the knee-point candidate is restricted to the cluster exhibiting the highest density around the trade-off region, typically characterized by intermediate objective values. Let $C_{j}$ denote the selected cluster. The final knee point is refined as:

(41) $$x^{*}=\arg\max_{x\in C_{j}}\kappa \left(x\right),$$

Hence, ensuring that the selected solution is not only geometrically optimal in terms of curvature but also statistically significant in terms of solution density [54]. The combined use of curvature-based analysis and GMM clustering provides a robust and physically interpretable mechanism for decision-making. It ensures that the selected operating point of the WPPVS corresponds to a high-probability, well-balanced compromise between economic cost and water supply reliability, rather than an isolated or unstable Pareto extreme.

### 2.7. Proposed Methodology

The developed WPPVS utilizes a GMM clustering-based MOPSO to identify the optimal size of the PV modules, HySS system, and storage tank. Equations (42)–(44), respectively, express the function (*J*), which is designed to minimize both the annual expenses of the system and the LWSP:

(42) $$J=min\;(f_{1},f_{2})$$

(43) $$f_{1}=\sum_{t\in T}^{T}LWSP\left(t\right)\;\;\forall \;t\in T$$

(44) $$f_{2}=\sum_{t\in T}^{T}\;C_{sys}\left(X_{t}\right)\;\;\forall \;t\in T$$

where, *T* is the set of hours per season and $X_{t}$ is the optimal solution, in which the near optimal decision variables are incorporated.

#### 2.7.1. Hourly Pumped Water

$S_{d}$ represents the hourly suction of water (pumped) from the well in metres is given as:

(45) $$S_{d}=\frac{Q_{d}\left(t\right)}{4\pi K_{com}H_{int}}W_{u}\left(t\right)$$

where, $K_{com}$ and $H_{int}$ are the hydraulic conductivity and the phreatic aquifer’s initial thickness, respectively. $W_{u}$ also known as the well function, is provided as [55]:

(46) $$W_{u}\left(t\right)=-0.5772-ln\left(U(t)\right)+U\left(t\right)-\sum_{n=2}^{\infty }\frac{{\left(-1\right)}^{n}\;U^{n}\left(t\right)}{n\times n!}$$

where, U(t) is given as:

(47) $$U\left(t\right)=\frac{R_{w}\;S_{c}}{4\;K_{com}\;H_{int}\;t}$$

where, $S_{c}$ is the ’storage coefficient’. The Appendix A section contains all of the parameters. In turns, the water plane ($W_{p}$) in the well is determined as:

(48) $$W_{p}\left(t\right)=H_{d}\left(t\right)-S_{d}\left(t\right)$$

with $W_{lev,th}$ demonstrated in Figure 1, set above 1m, the water plane in the well is confined by the following inequality:

(49) $$W_{p}\left(t\right)\geq W_{lev,th}$$

#### 2.7.2. Field Capacity

Weather data affect the wilting point ($W_{wp}$) in m^3^/h. As a result, it influences the hourly water need $M_{s}$, calculated from the water balancing requirements in (21), together with the ’dynamic peak field capacity’ ($W_{fc}$) in m^3^/h as:

(50) $$W_{wp}<M_{s}<W_{fc}$$

where,

(51) $$W_{fc}=\frac{A_{irr\;}E_{c}\left(t\right)\;W_{a}\;N_{plant}}{1000\;E_{u}}$$

(52) $$W_{wp}=0.25\;W_{fc}$$

$A_{irr}$ is the irrigated area, $W_{a}$ is the percentage of the region that is wet, $N_{plant}$ is the mean quantity of plants per hectare, $E_{u}$ is the percentage of drip system emission uniformity, $E_{c}$ is the evapotranspiration as in (20).

#### 2.7.3. Problem Formulation

**Figure 4:**
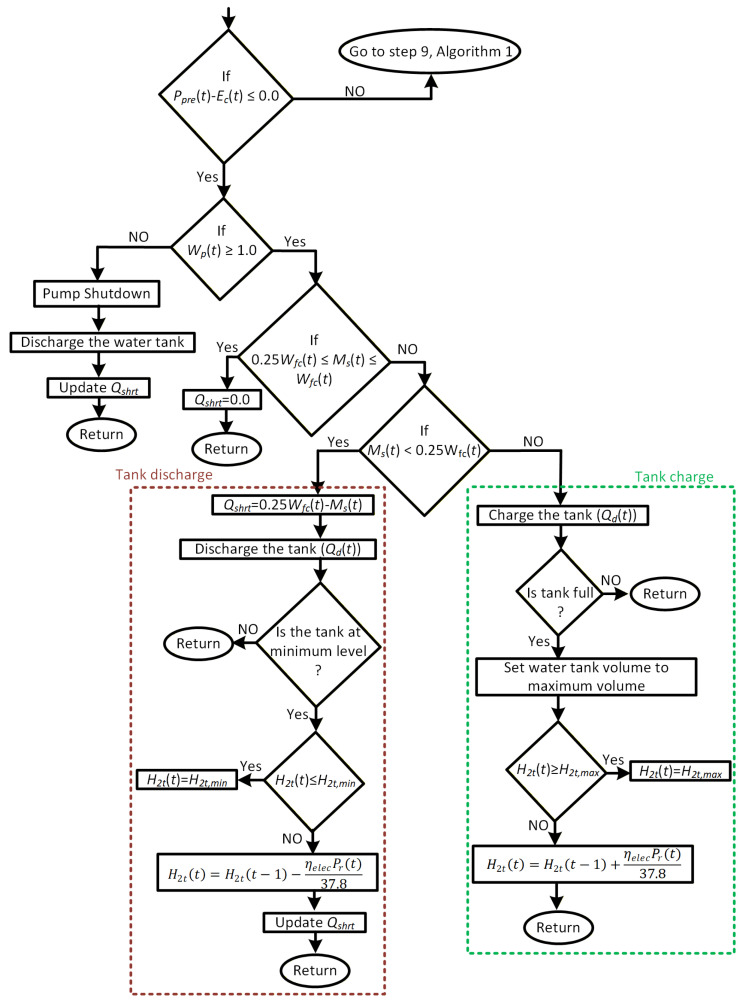
Charging/discharging modes of the WPPVS with HySS.

The overall EMS problem for the suggested WPPVS system is demonstrated in Figure 4 and the Pseudo-code is presented in Algorithm 1. As illustrated in Algorithm 1, the necessary amount of irrigation is calculated by considering historical data on wind speed, solar radiation, temperature, relative humidity, rainfall, and hourly precipitation. These historical data are employed in the developed system with the double storage of the water tank reservoir and the HySS to determine the energy flow. Therefore, the optimization is carried out using local meteorological data to determine seasonal energy costs. Precipitation ($P_{pre}$) falls behind evapotranspiration ($E_{c}$), depending ob whether the prescribed hydraulic demand constraints are satisfied or violated. The irrigation demand is thus required when the following restriction is met:

(53) $$E_{pre}\left(t\right)-E_{c}\left(t\right)\geq 0$$

If the energy production from the PV panel exceeds the water demand and the nonlinear characteristics of the pump are satisfied pursuant to the vibration avoidance settings, the extra energy is used to power the pump. Consequently, the water tank is charged and any leftover excess is then fed back into the HySS as Figure 4. The optimization strategy proceeds to meet the water needs throughout the crop season as shown in (50). The charging/discharging of the storage tanks aims to reduce the deficit ($Q_{shrt}$) in water needs from the storage tank as:

(54) $$Q_{shrt}\left(t\right)=W_{wp}\left(t\right)-M_{s}\left(t\right);\;\forall \;M_{s}<W_{wp}$$

When $M_{s}$ exceeds $W_{fc}$, the excess quantity of water is stored in the water storage tank, which is prioritized over hydrogen storage. Herein, the reliability index is the LWSP as in (55), which is a measure of the ability of the irrigation system to minimize the water shortage. LWSP ranges between 0 and 1, however, the WPPVS performs better when the LWSP is smaller.

(55) $$LWSP=\frac{\sum_{t=1}^{3672}\left(W_{wp}\left(t\right)-M_{s}\left(t\right)\right)}{\sum_{t=1}^{3672}W_{fc}\left(t\right)}$$

Table 2 presents the sensing infrastructure. This table specifies the sensor types and their technical characteristics (e.g., pyranometers for solar irradiation and piezoelectric Accelerometers for vibration). Additionally, the thresholds used (e.g., relative flow < 0.6) are directly linked to the output signals of these sensors, transitioning the model from theoretical inputs to sensor-derived data.

Primarily, Algorithm 1 is pivoted on optimizing the historical data to find the optimal values of the decision variables. To ensure the robustness of the proposed energy flow, a dedicated monitoring phase is proposed to tackle the influence of the measured or the estimated variables for the sensing and monitoring phase. The sensed variables are sampled at one-hour based intervals, while the estimated variables are updated from the historical data and the MOPSO results as well.
**Algorithm 1** Problem formulation modes of the developed WPPVS.  1: **procedure**
MOPSO(*k*, Hyper-parameters, meteorological data)  ▹*k* is the iteration number  2:      Initialize energy flow management phase (historical data)    ▹PHASE 1: ESTIMATION & CALCULATION  3:      Sk(1)←0.0     ▹Set initial values for the decision variables embedded in Equation (36)  4:      **for** k←1 to $N_{max}$ **do**      ▹$N_{max}$ is the maximum number of iterations  5:           $Q_{d}\leftarrow Equation\;\left(6\right)$  ▹The pump output flow rate according to Equation (6).  6:           $E_{c}\leftarrow Equation\;\left(20\right)$▹The actual evapotranspiration according to Equation (20).  7:           $P_{rain}\leftarrow Equation\;\left(22\right)$    ▹The estimated hourly rainfall according to Equation (22).  8:           $W_{fc}\leftarrow Equation\;\left(54\right)$  ▹The field water capacity according to Equation (51).  9:           $M_{s}\left(1\right)\leftarrow 0.0$, $W_{p}\left(1\right)\leftarrow W_{lev,th}$  ▹Set initial conditions for the moisture and the water levels10:           $M_{s}\left(t\right)\leftarrow Equation\;\left(21\right)$▹Calculate the hourly water needs balancing according to Equation (21).11:          $W_{u}\leftarrow Equation\;\left(46\right)$  ▹Calculate the water level in the well according to Equation (46).12:          $cost_{min}\leftarrow 10^{8}$13:          **if** 0.6<relativeflow<0.83 **then**       ▹according to Figure 2.14:               **if** $E_{pre}\left(t\right)-E_{c}\left(t\right)\leq 0$ **then**      ▹according to Equation (53).15:                    **Perform** the EMS in Figure 416:               **else**17:                    **goto** step 1018:               **end if**19:          **else**20:               **goto** step 521:          **end if**22:          $cost_{k}\leftarrow J_{k}\left(t\right)$▹$J_{k}\left(t\right)$ is the minimization function evaluated for *k* iteration as in Equation (42).23:          **if** $cost_{k}<cost_{min}$ **then**24:               $cost_{min}\leftarrow J_{k}$25:               $Sk\left(k\right)\leftarrow X_{t}$  ▹The optimal solution ever obtained from Equation (36)26:               MOPSO←Sk(k)   ▹Sk takes the corresponding value to *k*.27:          **end if**28:      **end for**29:      Input all non-dominated solutions from the Pareto archive into the GMM.30:      Assign solutions to n clusters based on the Gaussian probability density function.31:      Identify the optimal centroid by determining the optimal solution.    **return** MOPSO32:      Initialize sensing layer & monitoring (Flow, Level, Soil Moisture)    ▹PHASE 233:      Read real-time sensed Data: ($P_{actual}$, Dynamic head, water tank status, Meteorological data)34:      Ms(1)←0.0, $Wp\left(1\right)\leftarrow W_{lev,th}$35:      **Perform** the EMS in Figure 436:      Store the WPPVS performance diagrams37: **end procedure**

## 3. Simulated Results

This section investigates the optimization technique together with the GMM clustering on WPPVS using specific values of the dynamic head, crop selling prices, and economic factors such as interest and inflation rates. Appendix A provides the parameters of the developed optimization methods and the GMM clustering. The necessary parameters of the examined WPPVS and the pertinent crop prices were taken from [3]. IN addition, the HySS hyper-parameters were adopted from [30]. Sensitivity studies have been conducted to investigate the way the dynamic head, interest rate, and tomato prices affect the yield profitability. The developed MOPSO considering with vibration avoidance is used to determine the near optimal size of the hybrid storage WPPVS. The findings thereinafter could help farmers effectively use the WPPVS in the long-term to achieve appropriate performance in terms of seasonal costs and irrigation water utilization. The total seasonal costs and the LWSP are minimized together to find the near-optimal values of the decision variables (size of PV, volume of storage tank, number of hydrogen tanks, number of electrolyzers, and number of fuel cells) within a predetermined search space. A near-optimal centroid is thus obtained using the GMM clustering.

**Figure 5:**
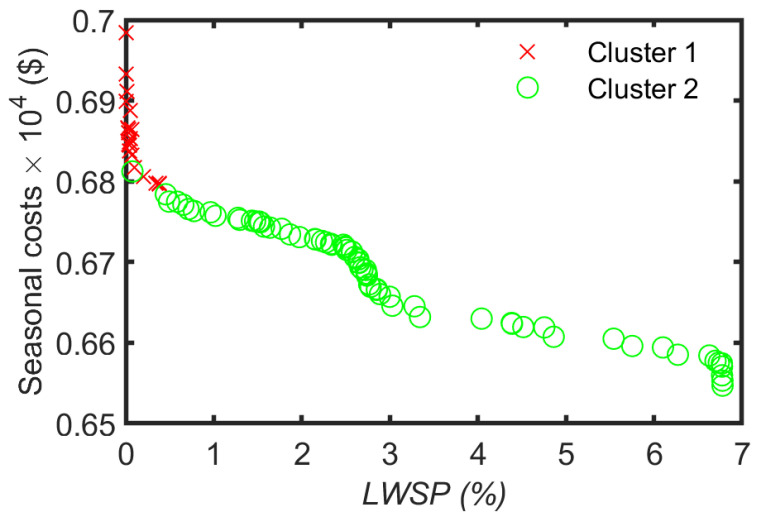
Pareto Front via GMM-based MOPSO.

Figure 5 presents the Pareto front obtained using the GMM-based MOPSO approach. To ensure a balanced optimization process, both objective functions are mapped into a comparable (dimensionless) range. The LWSP is inherently defined within the interval [0,1], as described in Equation (5). The seasonal cost, on the other hand, is scaled by a factor of $10^{4}$ (as shown in Figure 5), which normalizes its magnitude and improves numerical conditioning, although it does not strictly confine it to [0,1], but rather ensures comparable order of magnitude between objectives. Since LWSP and total seasonal cost are conflicting objectives, the resulting Pareto front represents a set of trade-off solutions rather than a single global optimum. Therefore, a knee-point selection criterion based on maximum curvature is employed to identify the most balanced compromise solution. The knee point corresponds to the region where a small improvement in one objective leads to a significant degradation in the other.

From Figure 5, the knee point is observed to lie within Cluster 2 (green points), which forms the well-distributed Pareto front. In contrast, Cluster 1 (red points) is concentrated near very low LWSP values but exhibits higher costs, and does not contribute to the main trade-off surface. It is important to note that these points are not strictly “dominated” in the Pareto sense, but rather represent a less favorable region of the solution space with poor trade-off characteristics. The selected operating point of the WPPVS corresponds to approximately LWSP ≈ 2.5–3%, which lies near the region of highest curvature. To the right of the knee, further reductions in cost come at the expense of a rapid increase in LWSP, indicating reduced system reliability. Conversely, to the left of the knee, achieving marginal improvements in LWSP requires a disproportionately large increase in cost. This confirms that the knee point represents the most economically efficient and operationally balanced configuration. The observed trade-off between Objective 1 (total cost) and Objective 2 (LWSP) arises because improving water supply reliability (lower LWSP) typically requires increased system capacity, redundancy, or operational intensity, all of which raise costs. The GMM clustering highlights that Cluster 2 captures the primary Pareto trade-off region, while Cluster 1 corresponds to a boundary region with limited practical relevance for decision-making.

The primary objective of this study is the techno-economic feasibility and capacity sizing of the WPPVS (Phase 1). This optimization is indeed driven by historical datasets to establish the baseline configuration presented in Table 3. Regarding Real-Time Performance Evaluation (Phase 2), sensing serves as an Open-Loop Monitoring System rather than a Closed-Loop Control. Dynamic Variables such as Solar irradiation, wind speed, and flow rates are sensed in real-time to estimate instantaneous system performance and efficiency. Parameters such as vibration and soil moisture are currently utilized for condition monitoring and safety thresholds (e.g., preventing pump vibration or over-irrigation) rather than as feedback variables variables in a closed loop control fashion.

### 3.1. Coordinated WPPVS

**Figure 6:**
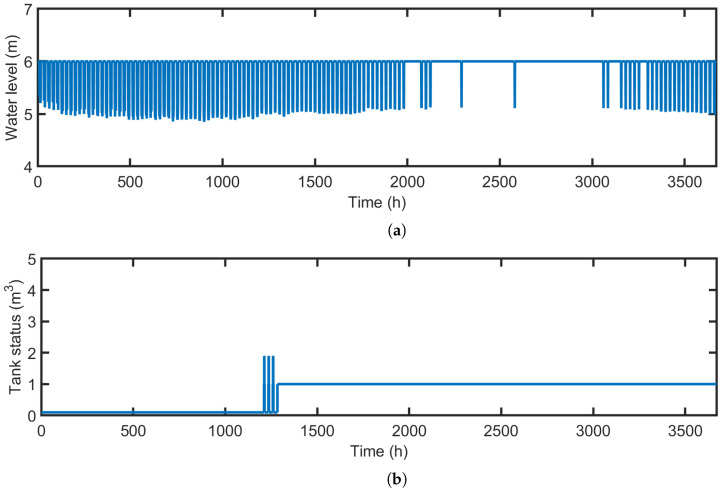

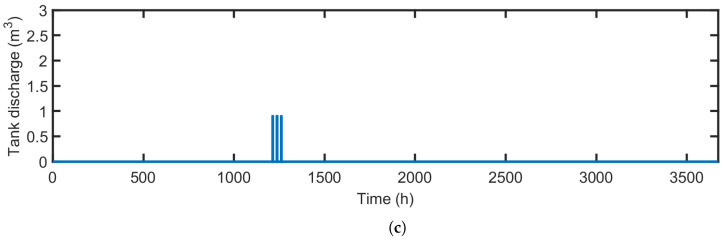
Performance at the near-optimal solution. (**a**) Water level performance. (**b**) Status of the tank. (**c**) Tank discharging flow.

Remarkably, the optimal point in the computed Preto-front masks the optimal choice variables. Table 3 shows the near-optimal solution of the decision variables assuming the PV and fuel cell sizes are taken into account as multiples of 50 W. Since finding fractions is bizarre, the 50 W was chosen to reflect the real-world field ratings. Consequently, the LWSP values and seasonal costs are determined to be 2.6% and 7.91 k$, respectively. As illustrated in Figure 6a, the pump runs for 347 h during the irrigation season with a dynamic water head of 6m. Additionally, the water obtained by employing HySS storage amounts to 7355 m^3^. This is compared to 527 h for pump operation and 10,220 m^3^ water extraction without HySS [3], which reduces the water pumping hours and thus preserves a satisfactory underground water level. In the middle of the tomato season as shown in Figure 6b and Figure 6c, respectively, when there is a significant demand for water, the outflow rate of the water tank is exceptionally high.

### 3.2. Impact of the HySS on the Use of Irradiation

**Figure 7:**
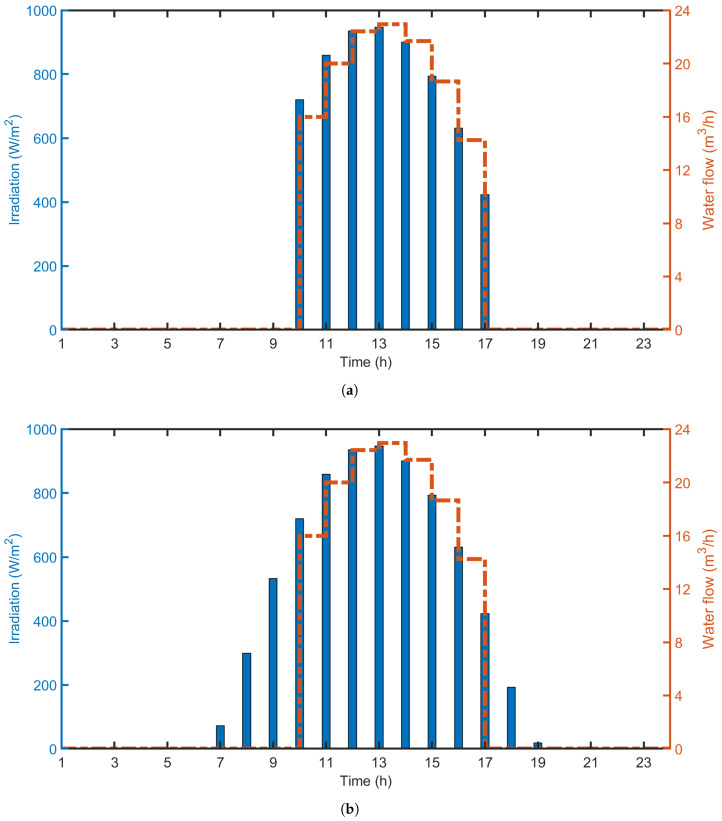
HySS influence on irradiation utilization. (**a**) Without HySS. (**b**) With HySS.

When evaluating the HySS performance, it is important to factor the the number of hours the WPPVS is on. In Sakaka, irrigation takes only 7 h with employing the pump vibration avoidance strategy, compared to 12 h when using the pump endures vibrations. As seen in Figure 7a, this reduces the exploitation of the per-day use of solar irradiation. Consequently, using the developed energy coordination and against 1071 h without HySS, the near-optimal design of the WPPVS reveals that the seasonal irradiation harvest hours are 1863. The augmentation of the HySS increases thus the use of the solar irradiation hours by 5 h, which represents 21% (=$\frac{5}{24}\times 100$) of the seasonal hours as in Figure 7b.

### 3.3. Seasonal Energy Mapping of an Integrated the WPPVS System

**Figure 8:**
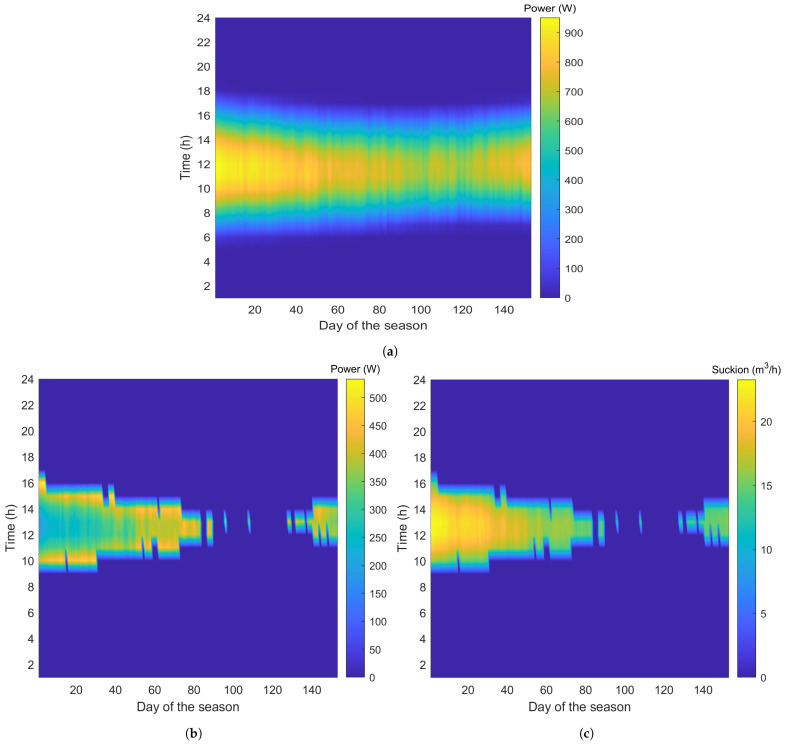
WPPVS augmented HySS energy operation. (**a**) PV hourly operation. (**b**) Fuel cell hourly operation. (**c**) Pump operation mode.

Figure 8 reveals the relationship between the available solar energy and the operational decisions of the fuel cell and the pump based on the results of phase 2 of Algorithm 1. The three-panel analysis demonstrates a clear hierarchical dependency within the system. Figure 8a (PV Power) illustrates the maximum available energy envelope, showing a broad solar window stretching from 06:00 to 18:00 with peak intensities reaching 900 W. In contrast, the fuel cell and the pump operations (Figure 8b and Figure 8c, respectively) are nested within this window but are significantly more constrained, operating only between 10:00 and 16:00. While PV power is available for approximately 12 h a day, the fuel cell and the pump are activated only during the hours of peak solar irradiation. This suggests a power-density threshold where the system only engages when solar generation exceeds approximately 600 W (as seen by comparing the yellow zones in Figure 8a with the start times in Figure 8b and Figure 8c, respectively). The intensity of operation shows a distinct seasonal trend: during the first 80 days, the system maintains peak performance, with the fuel cell generating approximately 450–500 W to support a suction rate exceeding 20 m^3^/h. However, between day 80 and day 130, there is a visible reduction in both the duration and magnitude of operation, characterized by more fragmented activity and lower power levels. This pattern likely reflects fluctuating energy availability or varying hydraulic demand across the season. For approximately the first 40 days, the pumping mode operation runs from 10:00 to 16:00 pm, and for the remaining 40 days, it runs from 11:00 to around 15:00. As a result, fuel cell operation is notable during these extreme hours of these periods.

The fuel cell power peaks at 500 W, which is roughly half of the maximum PV output. This is because the system is designed to either store excess solar energy in the hydrogen tank and the fuel cell acts as a stabilized power conditioner to ensure that the pump receives a steady, high-quality current of 500 W to maintain a suction rate of 20 m^3^/h. Figure 8 shows a corresponding “dip” or narrowing of the power band between Day 80 and Day 120. Because this trend is visible in the PV heatmap as shown in Figure 8, it confirms that the reduced performance in the fuel cell and pump during this period is a direct result of lower solar insolation. This is likely due to the winter months or seasonal rainfall.

### 3.4. Sensitivity Analysis

**Figure 9:**
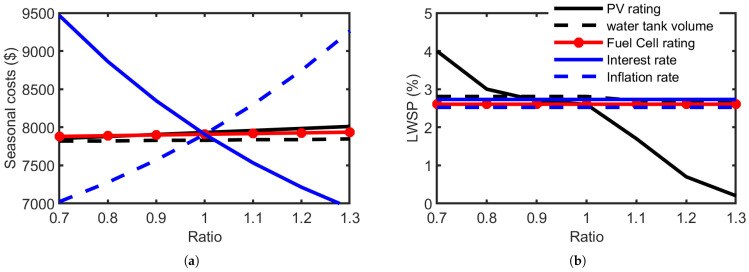
Sensitivity analysis upon the WPPVS. (**a**) Impact on the seasonal costs. (**b**) Impact on the LWSP.

**Figure 10:**
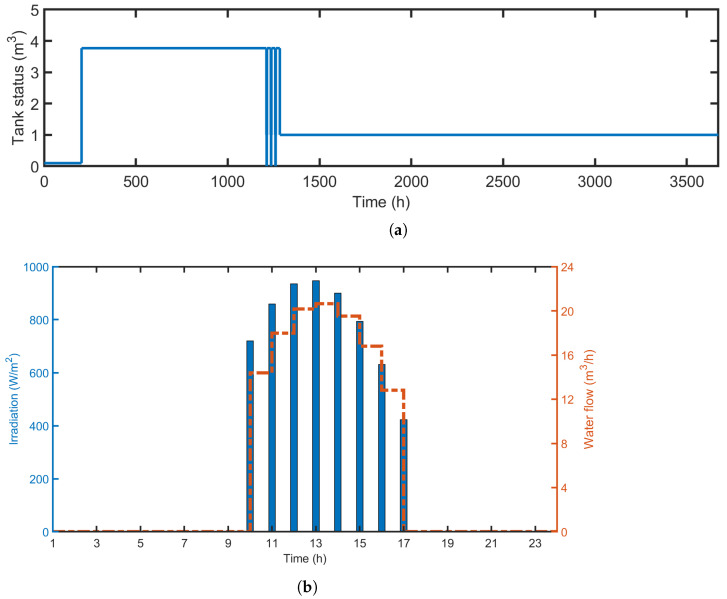

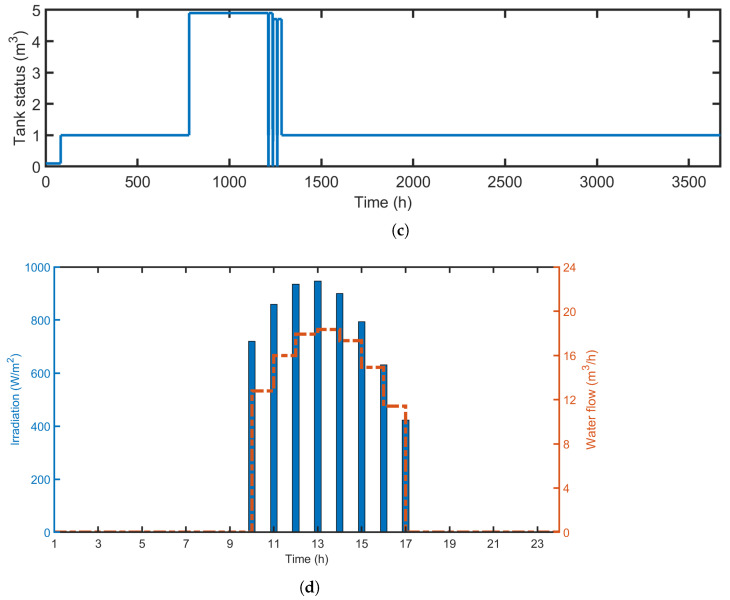
Impact of PV rating on tank status and daily pumped water. (**a**) Tank status at 0.9 of the optimal PV rating. (**b**) Pumped water at 0.9 of the optimal PV rating. (**c**) Tank status at 0.8 of the optimal PV rating. (**d**) Pumped water at 0.8 of the optimal PV rating.

In this section, the effects of PV sizing, water tank capacity, HySS fuel cell sizing, and interest and inflation rates on the seasonal expenses, LWSP, and daily pumped water are examined. The nominal values of the near-optimal parameters are modified by ±30%, while the other parameters are kept unaltered. In Figure 9, the PV demonstrates a minor variation in seasonal costs but a substantial nonlinear propensity to change in the LWSP. With a higher PV rating energy, the LWSP performs better, reaching practically acceptable values of less than 1%. While they have little effect on the LWSP, interest and inflation rates have a significant impact on seasonal costs. As the interest rate rises, session expenses decrease, while inflation drives up seasonal prices. As the interest rate rises, the session costs decrease, while inflation pushes seasonal costs higher. In Figure 10, the near-optimal PV is changed to consider its influence on the daily pumped water and the storage of the water tank. Decreasing the nominal size of the PV increases the water volume of the water tank. In contrast, it significantly decreases the daily injected water.

### 3.5. Findings and Discussion of the Implications for Practical Engineering

The integration of the MOPSO-GMM framework provides actionable insights into the operational management of WPPVS. Practically, the identified Pareto clusters allow farmers to transition from a single-point deterministic solution to an image-aware vision. For instance, during periods of high rains, operators have the chance to shift the system state toward the ’Hydrogen production’ identified by the supervisory control unit and the weather station as shown in Figure 1 to mitigate mechanical fatigue on the pump vibrations. Furthermore, the probabilistic nature of the GMM framework serves as a visual diagnostic tool; the overlap between Gaussian components indicates a ’satisfactory zone’ where the system can absorb fluctuations in water inflow or solar irradiation without requiring a re-optimization of the entire dispatch schedule. This reduces the computational burden on real-time SCADA systems, making the proposed approach highly suitable for integration into an automated EMS for large-scale renewable integration.

The practical implications of the proposed framework are summarized in Table 4, which evaluates system performance across five distinct techno-economic scenarios. Under extreme economic conditions, such as high interest rates and low inflation (−11.5% cost variance), the system maintains a low LWSP stress, thereby proving the robustness of the optimization. This effect arises from the discounting mechanism in the economic model, where higher discount rates reduce the present value of future capital and replacement costs. The GMM-optimized knee point provides the most reliable configuration for standard operations, achieving a ’balanced’ stress level of 2.6%. Furthermore, the sensitivity analysis on PV sizing reveals that while over-sizing offers marginal reliability gains, whereas down-sizing the PV by 30% significantly compromises the system (increasing stress to 4%), thus identifying the critical threshold for system viability.

**Figure 11:**
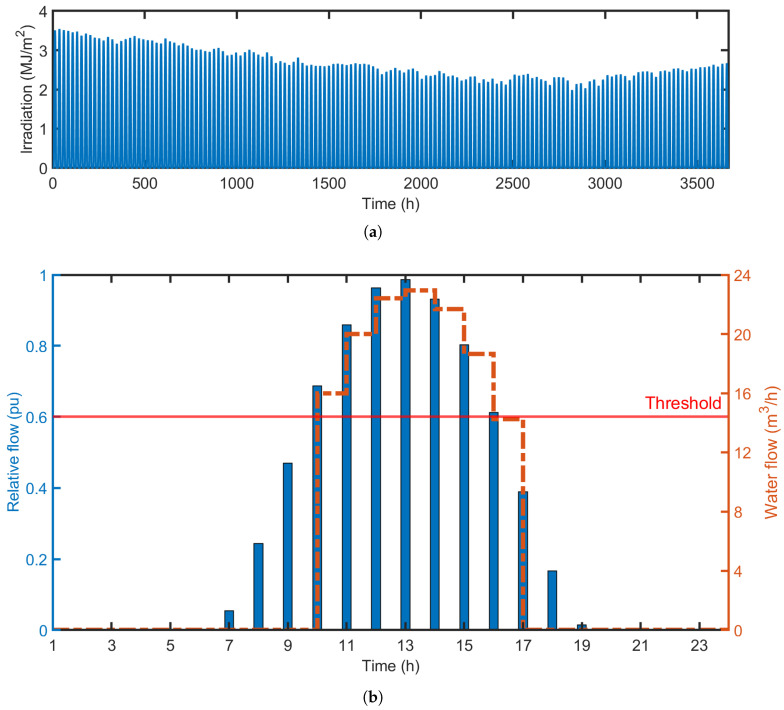
Performance monitoring of the WPPVS. (**a**) Solar irradiation. (**b**) Vibration avoidance strategy performance.

The solar irradiation is shown in Figure 11a, which directly influences the pumped water. There is a clear macroscopic trend where the peak irradiation gradually decreases from t = 0 (starting around 3.5 MJ/m^2^) toward t = 2500 (dipping near 2.0 MJ/m^2^), before beginning a slight upward trend toward the end of the data set. The water level in the well as in Figure 6a varies between 5m and 6m. This limited head variation ensures that the hydraulic characteristics do not shift drastically, allowing a relative flow rate threshold of 0.6 to remain a reliable indicator of the onset of vibration.

- Argumentation in Sensing (The 0.6 Threshold): Figure 11b, demonstrates the practical application of the Qref < 0.6 threshold. As shown at t = 17h, when the relative flow (pu) drops below the threshold due to decreasing solar input, the system control logic successfully initiates a reduction in water flow (m^3^/h) to avoid the high-vibration regime.
- Hysteresis and Control Logic: The control logic is (0.6 < Qref < 0.83) to avoid the pump vibration. Looking closely at the difference between Hour 9 and Hour 17. In Hour 9, the relative flow is lower than 0.5, but the system is just starting up. In Hour 17, it is dropping.

To sum up, the engineering significance of the results shown in Figure 11 is twofold. First, the solar profiles (a) provide the boundary conditions for system reliability, necessitated by the stochastic nature of the energy input. Second, the threshold-controlled flow in (b) serves as a protective measure for the mechanical infrastructure. By restricting pump operation to ≥0.6 pu, the system avoids the high-vibration regions identified in Figure 2, effectively translating algorithmic optimization into reduced mechanical fatigue and enhanced system robustness.

A limitation of the current control logic is the use of a static threshold for pump activation. While the 0.6 pu limit protects the system from low-efficiency operation, it does not currently account for potential ’chattering’ effects during transient cloud cover, which requires designing linear-time state space variable model (via the differential equations). Implementing a hysteresis band—where the system activates at 0.6 pu but remains operational until a lower ’deactivation’ limit of 0.55 pu is reached—would further stabilize the mechanical response, though it was outside the scope of this initial optimization.

## 4. Conclusions and Perspectives

This study presented a robust multi-objective optimization framework for water pumping photovoltaic systems (WPPVS) augmented hydrogen energy storage system (HySS), integrating MOPSO with a Gaussian Mixture Model (GMM) to address the inherent conflicts between total costs and the loss of water supply probability. By shifting from a purely deterministic approach to a GMM-based one, the proposed framework successfully identified ’optimal solution’ within the Pareto Front that remains valid under the variations in water demand. The practical implications of this research are twofold: first, the GMM clusters provide a simplified solution map for WPPVS operators, allowing for satisfactory trade-off between ’Economic’ and ’water loss’ without re-optimization. Second, the sensitivity analysis confirmed that the framework maintains robustness quality even during ±30% change in the system parameters. The suggested approach allows pre-mapping of WPPVS augmented HySS energy operation in terms of hydraulic pump characteristics, irrigation water balance, crop yield response, hydrogen generation and storage dynamics, and economic assessment into a single optimization process rather than depending on oversimplified sizing assumptions. To increase efficiency and lessen vibration-induced mechanical stress, pump operation was restricted to remain within a stable range in relation to the optimal efficiency point. Based on the results and assessments, the following conclusions can be drawn: (1) The application to a tomato drip irrigation system in Sakaka, Saudi Arabia demonstrated that coordinated management of photovoltaic generation, water storage, and hydrogen storage enhances system reliability and operational flexibility, (2) The Pareto-front analysis and knee-point selection yielded a balanced operating solution achieving a low LWSP of approximately 2.5–3% while maintaining competitive seasonal costs, (3) Sensitivity analyses further indicated that the selected design remains robust under variations in hydraulic conditions, crop market prices, and economic parameters, confirming its resilience to realistic technical and financial uncertainties. The performance of the LWSP and daily pumped water are significantly affected by the PV size. Meanwhile, seasonal costs seem to be significantly influenced by interest and inflation rates. (4) The daily enhancement of the HySS increases the use of solar irradiation hours by 21% of the seasonal hours, which could be used for the long-term production and trading of renewable hydrogen. (5) The developed WPPVS is both reliable and user-friendly, and it has the potential to be tested with a variety of hybrid storage systems.

Future work will focus on integrating real-time weather forecasting data into the GMM priorities to further refine the predictive methodology of the energy dispatch between the PV and the fuel cells under extreme climate events. In addition, to assess operational coordination, dependability, and economic viability, a pilot-scale study (e.g., a 2-hectare farm) will be conducted, considering many crops to get the necessary characteristics. Regarding the development of a real-time sensor-driven control loop, via utilizing active feedback from vibration and flow sensors, the system can transition from open-loop monitoring to a dynamic response closed-loop control. This approach is expected to further improve system reliability and energy utilization by implementing sophisticated hysteresis bound for pump activation and shutdown.

## Figures and Tables

**Table 1:** Compact summary of WPPVS literature survey.

Method	RES	Storage (B/T/H)	Load to be Served	Vibration Avoidance
NSGA-III [3]	PV	C/C/NC	Irrigation	C
Analytical [16]	PV	NC/NC/NC	Irrigation	NC
Analytical [17]	PV	C/C/NC	Irrigation, Cattle	NC
HPPT [18]	PV	NC/NC/NC	Water pumping	NC
Analytical [19]	PV	C/NC/NC	Irrigation	NC
P&O [33]	PV	C/NC/NC	Water pumping	NC
PHOTOV-IV [34]	PV	C/NC/NC	Pumping, Electric loads	NC
Dynamic model [35]	PV	NC/NC/NC	Irrigation	NC
Analytical [20]	PV	NC/NC/NC	Irrigation	NC
Mathematical [36]	PV	NC/NC/NC	Water pumping	NC
HOMER opt. [21]	PV	C/NC/NC	Irrigation	NC
PVsyst opt. [37]	PV	NC/C/NC	Drip irrigation	NC
Analytical [22]	WT + PV	NC/C/NC	Water pumping	NC
AI [23]	WT + PV	C/C/NC	Irrigation	NC
Analytical [38]	PV	NC/NC/NC	Irrigation	NC
Analytical [39]	PV	NC/NC/NC	Irrigation	NC
Analytical [40]	PV	NC/NC/NC	Irrigation	NC
Analytical [41]	PV	C/NC/NC	Irrigation, Power gen.	NC
Equilibrium opt. [10]	PV	C/NC/NC	Irrigation	NC
NSGA-II + MOPSO [42]	WT + PV	NC/NC/NC	Irrigation	C
Fuzzy logic [43]	PV	C/NC/NC	Water pumping	NC
Analytical [44]	NC	NC/NC/NC	Water pumping	NC
Analytical [45]	PV	C/NC/NC	Irrigation	NC

WT: Wind turbine, B: Battery, T: Tank, H: Hydrogen storage, C: Considered, NC: Not considered.

**Table 2:** WPPVS sensing infrastructure.

Variable	Proposed Sensor Type	Specifications	Role in the System
Solar irradiation	Pyranometer (e.g., ISO 9060 Class A)	Spectral range: 285–3000 nm	Input for energy availability
Flow rate	Ultrasonic flowmeter	Accuracy: ±1%; non-invasive	Monitoring fluid dynamics
Vibration	Piezoelectric Accelerometer	Frequency range: 10 Hz–10 kHz	Detecting mechanical instability
Soil moisture	TDR (Time Domain Reflectometry)	Operating range: 0–100% VWC	Feedback for irrigation control
Meteorological data	Integrated weather station	Wind speed, humidity, temp	Environmental context

**Table 3:** Near-optimal WPPVS sizing.

PV (W)	Water Tank (m^3^)	HySS		
		Fuel Cell (W)	Number of Tanks	Number of Electrolyzers
1150	5	500	1	1

**Table 4:** Practical engineering implications.

Condition	Cost Increase	LWSP Stress	Primary Use Case
Low interest rates (−30%) high inflation rate (+30%)	+20.1%	<2.6%	Advanced countries
High interest rates (+30%) low inflation rate (−30%)	−11.5%	<2.6%	Developed countries
GMM-optimized knee	optimized	balanced (2.6%)	Standard daily operation for developed countries
Over-sizing the PV (+30%)	insignificant	LWSP is low	High-groundwater regions
Down-sizing the PV (−30%)	insignificant	LWSP is moderate (4%)	Sub-optimal operation

## Data Availability

The data presented in this study are available upon request from the corresponding author.

## Supplementary Materials

The following supporting information can be downloaded at: https://www.mdpi.com/article/10.3390/s26113350/s1, Table S1: List of Symbols.

## Abbreviations

BEP	Best Efficiency Point
EMS	Energy Management System
ESS	Energy Storage System
FAO	Food and Agriculture Organization
GMM	Gaussian Mixture Model
HySS	Hydrogen Energy Storage System
LWSP	Loss of Water Supply Probability
NPC	Net Present Cost
MOPSO	Multi-objective Particle Swarm Optimization
PV	Photovoltaics
RES	Renewable Energy Resources
WPPVS	Water Pumping Photovoltaic System

## Appendix A. Optimizers’ Setup Parameters

**MOPSO:** population = 100, Repository Size = 20, ω = 0.1, c_1_ = 0.25, c_2_ = 0.99, r_1_ = 0.3, r_2_ = 0.45, α = 0.1, β = 0.2, γ = 0.2, μ = 0.001, Number of Grids per Dimension = 5, number of iterations = 50.

**GMM:** number of clusters = 2, gmmModel = fitgmdist.

**Hydrogeological Clarity:** The permeability coefficients and aquifer properties used in well-level calculations that have been added to the technical supplement to ensure full replicability. Hence, the permeability coefficients and aquifer properties used in the well-water level calculations have been stated in the Technical Supplement. Furthermore, the operational parameters for the examined (WPPVS) and the relevant crop prices were adopted from [3].
