# Supplementary material for: Optimal Techno-Economic Feasibility of Solar PV Irrigation System Augmented Hydrogen Energy Storage
Source: Sensors (Basel). 2026 May 25;26(11):3350. doi: 10.3390/s26113350 (PMC13259112; doi:10.3390/s26113350)
Supplement: Supplementary file 1 [file sensors-26-03350-s001.zip › sensors-4181855-supplementary.pdf]

Table S1. List of symbols

A	Irrigation area (ha)
C_{in}	Seasonal revenue from tomato crop ($)
C_{sys}	Total cost of WPPVS ($)
C_{agr}	Seasonally agricultural costs ($)
C_{ic}	Component initial cost ($/kW)
C_{repc}	Replacement cost of a component ($/kW)
C_{omc}	Component operating and maintenance costs ($/kW)
C_{salvc}	Component salvage cost ($/kW)
e_s	Vapour pressure at Saturation (kPa)
e_a	Actual hourly vapour pressure (kPa)
E_c	Hourly actual evapotranspiration (m^3/h)
E_{fc}	Fuel cell output power (kW)
E_{ref}	Hourly reference evapotranspiration (mm/h)
E_u	Emission uniformity percentage of drip system
f_{PV}	Derating factor of PV module
g	Gravitational field strength (N/kg)
G	Hourly soil heat flux density (kWh/ m^2)
$G_n(t)$	Solar radiation (kWh/m^2)
H_{2p}	Hydrogen produced by the electrolyzer (kg)
H_{2t}	Hydrogen accumulated in the hydrogen tank (kg)
H_t	Total water head (m)
H_d	Initial value of the dynamic head (m)
H_{int}	Initial thickness of the phreatic aquifer (m)
U_{2u}	Hydrogen used (kg)
K_c	Crop coefficient
K_e	Emission factor (kg/L)
K_y	Yield response factor
K_{con}	Hydraulic conductivity (m/h)
LF_c	Component life time (years)
LWSP	Loss of water supply probability
$M_s(t)$	Hourly value of the soil moisture at each hour t (m^3/h)
NPC_c	Net present cost of each component ($/kW)
N_{plants}	Average number of plants per hectare
$P_{PV}(t)$	Hourly output power from the PV generator (kW)
P_{PV_rat}	Nominal power of PV generator (kW) at standard operating condition
P_{hdro}	Hydro power output from the pump (W)
P_{pre}	Precipitation (m^3/h)
P_r	PV output power at the electrolyser side (kW)
P_{rain}	Rainfall (m^3/h)
p_t	Price of tomato ($/ton)
Q_d	Water flow rate (m^3/h)

Q_{dmax}	Maximum possible pumped water volume per hour (m^3/h)
R_w	Radial distance from pumped well (m)
S_c	Storage coefficient
$S_d(t)$	Hourly suction of water from the well at each hour t (m)
$T_{air}(t)$	Ambient air temperature at each hour t (ºC)
$T_c(t)$	Cell temperature of PV module at each hour t (ºC)
T_{tran}	Transmissivity coefficient (m^2/h)
v_w	Hourly wind speed at 2 m height (m/s)
W_{req}	Hourly water requirement (m^3/h)
W_{fc}	Dynamic peak field capacity (m^3/h)
W_{wp}	Welting point (m^3/h)
W_a	Percentage of wetted area
$W_{lev}(t)$	Hourly water-level in the well at each hour t (m)
$W_{lev,th}$	Minimum permissible (threshold) water- level (m)
W_u	Well function
Y_a	Actual crop yield Y_a (ton /ha)
Y_g	Intercept coefficient of fuel curve (L/kWh)
Y_m	Maximum crop yield (ton /ha)
ρ_w	Water density (kg/m^3)
η_p	Efficiency of water pump
η_{inv}	Efficiency of inverter
Δ	Slope of vapour pressure curve (kPa/ °C)
γ	Psychrometric constant (kPa/ °C)
ρ_w	Water density (kg/m^3)
η_{FC}	Fuel cell efficiency
η_{elec}	Electrolyzer efficiency
η_p	Efficiency of water pump
